# Green sustainability in the hotel sector: The role of CSR, intrinsic green motivation, and personal environmental norms

**DOI:** 10.1371/journal.pone.0295850

**Published:** 2024-06-27

**Authors:** Zhihong Meng, Saad Mahmood Bhatti, Rana Tahir Naveed, Sara kanwal, Mohammad Adnan

**Affiliations:** 1 General Education Department, Hebel Tourism College, Chengde, China; 2 Institute of Business and Management (IB&M), University of Engineering and Technology (UET), Lahore, Pakistan; 3 Graduate School of Business (GSB), Universiti Kebangsaan Malaysia (UKM), Selangor, Malaysia; 4 Division of Management and Administrative Sciences, University of Education (UE) Business School, University of Education, Lahore, Pakistan; 5 Business and Management Department, SBS Swiss Business School, Kloten, Switzerland; National University of Modern Languages, PAKISTAN

## Abstract

In the context of the United Nations Sustainable Development Goals (UN-SDGs), this study accentuates the role of the tourism and hospitality sector in promoting sustainability. The primary purpose is to unravel the relationship between corporate social responsibility (CSR) and energy-specific sustainable behavior of employees (ESBE), with particular emphasis on the mediating roles of green intrinsic motivation and personal environmental norms. Utilizing a three-wave data collection approach, we secured 325 valid responses from sector employees at various levels (manager-non managers) and applied Structural Equation Modeling through the SMART-PLS tool to assess the hypothesized relationships. The findings highlight a pronounced interconnection between CSR, ESBE, and the designated mediating variables. These results not only augment the academic literature by illustrating the psychological underpinnings bridging CSR to ESBE, but also equip the tourism and hospitality industry with actionable insights. Through informed CSR initiatives aligned with employee values, the sector can galvanize sustainable behaviors and create business models that resonate with the aspirations of the UN-SDGs, pointing the way to a more sustainable industry.

## 1. Introduction

Climate change has profound effects on our ecosystems, extending beyond weather variations to rising sea levels and affecting human existence [1]. The need for action is crucial not just for governments but also for local communities and individuals [2]. A transition to a carbon-neutral world requires cooperation across sectors, emphasizing renewable energy, sustainable practices, and fostering an environment-conscious global culture [3]. The United Nations Sustainable Development Goals (UN-SDGs), with 17 interlinked objectives, address challenges from poverty to peace, with several goals concentrating on environmental protection and climate change [4]. Goal 13 calls for urgent action against climate change, while Goal 7 promotes affordable, clean energy. Goals 11 and 15 also touch on sustainability [5]. Modern businesses increasingly align with the UN-SDGs, with employees playing a key role in implementing these alignments [6]. Their commitment to energy efficiency and sustainability programs is vital for environmental sustainability [7]. Goal 8 underscores the value of a sustainability-supportive workplace.

Individual sustainable actions are broadly termed as pro-environmental behavior (PEB). Kollmuss and Agyeman define PEB as behaviors reducing one’s environmental impact [8]. In organizations, PEB includes practices like using scrap paper, bringing reusable items, and conserving resources [9]. Much research has discussed PEB broadly, but there is a growing need to focus on energy-specific sustainable behavior of employees (ESBE), which deals with individual energy consumption patterns in offices [3, 10]. Enhancing ESBE represents a company-wide dedication to sustainability.

Corporate social responsibility (CSR) initiatives, especially those targeting ecological preservation, can bolster the responsible behaviors of individuals [11, 12]. When organizational values and CSR align with individual behaviors, a culture prioritizing sustainability emerges [13, 14]. Participating in renewable energy programs or waste reduction efforts, for example, signals the importance of sustainable practices to employees. These initiatives, combined with ESBE-supportive policies, can enhance responsible energy use [15]. However, the relationship between CSR and individual behavior is sophisticated [16, 17], as personal and psychological aspects mediate this relationship [18]. These factors bridge organizational values expressed via CSR to individual actions [19]. Their presence helps us understand how CSR can influence personal behaviors in varied situations. CSR indicates a business’s commitment to ethical conduct and environmental stewardship [20, 21]. Transitioning CSR towards ESBE is not just about implementing policies; it involves understanding the psychological factors driving these actions.

The literature suggests that green intrinsic motivation [22, 23] and employees’ personal environmental norms [24, 25] mediate responsible environmental behaviors. While the convoluted connection between CSR and personal behavior is acknowledged, the mechanism driving ESBE within a CSR framework remains unclear. An exploration of organizational values offers insights into a firm’s strategic compass while exploring individual values shines light on the foundational attitudes that influence day-to-day operations. The interplay between these two layers is vital for a comprehensive understanding of sustainability integration. We aspire to decipher this by examining green intrinsic motivation and personal environmental norms in the CSR-ESBE nexus. Our aim is to understand how these elements influence ESBE within CSR, shedding light on the intricate bond between individual behaviors and CSR.

Green intrinsic motivation originates from personal beliefs, prompting individuals to act responsibly [26]. Unlike external motivators like money, this motivation stems from inner principles. When driven by an innate desire to benefit the environment, employees willingly adopt sustainable practices, enhancing a company’s CSR impact on ESBE. Similarly, personal environmental norms, influenced by upbringing, culture, and education, guide employees toward sustainability [27]. When these align with CSR goals, employees actively support sustainability, potentially leading to a more profound realization of ESBE.

Uniquely, this study differs from previous work due to its focus on the specific issues facing Pakistan’s ever-changing environment within the framework of evolving social and economic dimensions [28, 29]. Most of the earlier researches were centered on CSR in a developed or western context. Ours focuses on CSR in developing Pakistan’s tourism sector. One important aspect of Pakistan’s economy is hardly studied concerning sustainability as well as CSR and ESBE. This study provides an insight into emotional determinants of green intrinsic motivation and personal internalized regulations towards the environment among a Pakistani community. Given the culture, education, society, and other factors that inform employees’ attitudes and behaviors on sustainability in Pakistan, this focus is quite relevant [30]. These subtle differences, as we ignore them at a bigger international perspective of global studies, become imperative while devising appropriate CSR mechanisms to work in Pakistan. Finally, our study links global sustainability frameworks like UN-SDG’s with its practical use to Pakistani setting. This paper addresses the integration of these global objectives into the business plans for Pakistan’s tourism and hotel sector, in consideration of its unique requirements and constraints.

Our choice of respondents, primarily employees within this sector, was deliberate. Employees act as bridges between high-level organizational strategies and ground-level execution. Their perceptions, motivations, and behaviors directly influence the realization of sustainability objectives, making them indispensable to any discourse about CSR and sustainable practices. By concentrating on this demographic, we sought to unravel the interplay between individual psychological elements and broader organizational directives, with the ambition of unveiling actionable insights for companies to foster a more sustainable work ethos. Yet, the literature has seldom addressed this intricate dance between employee behaviors, corporate strategies, and global frameworks in such a context. This evident gap underscores our study’s importance and urgency.

This study tends to explore the interplay between CSR, green intrinsic motivation, personal environmental norms, and ESBE in Pakistan’s tourism and hospitality sector. This sector’s selection is pivotal for numerous compelling reasons. Globally, the tourism and hospitality industry leaves a substantial environmental mark, characterized by its extensive resource consumption which in turn leads to escalated emissions and waste [31–33]. Pakistan, as underlined by recent reports from esteemed bodies such as the World Bank Group and Asian Development Bank and others, stands at a unique crossroads of climate vulnerabilities and socio-economic aspirations [34–37]. This intricate balance underscores the urgency of sustainable interventions, particularly in pivotal sectors like tourism and hospitality. In Pakistan, the growing growth of this industry, juxtaposed against the backdrop of rapid urbanization, magnifies these environmental challenges. The exploration of CSR’s role and ESBE within this context becomes both pressing and opportune. Furthermore, this emphasis is harmonious with several UN-SDGs, notably Goal 7, Goal 12, and Goal 13, emphasizing the importance of sustainability and environmental conservation. In diving deeper into the psychological aspects like green intrinsic motivation and personal environmental norms within this sector’s workforce, the study aims to unearth how individual principles can align with broader organizational objectives, potentially driving more responsible energy consumption behaviors. The overarching objective is to discern ways for tourism and hospitality entities to minimize their energy footprint through strategic employee engagement, a quest of paramount importance in a country where energy conservation is crucial. By zeroing in on Pakistan’s tourism and hospitality sector, this research not only underscores the industry’s economic significance but also its profound potential for environmental sustainability. The insights gleaned from this context will invariably enrich our understanding of how CSR, aligned with employee psychology and sustainable practices, can resonate with global sustainability aims. This knowledge holds promise for guiding industries both within Pakistan and globally as they grapple with the delicate dance of growth and environmental responsibility.

Our study makes several key contributions to the existing body of research on sustainability, especially within a developing country milieu such as Pakistan. The main contribution is its focus on CSR and ESBE for tourism and hospitality industry. While there are few studies that address specific sectors within developing countries [38, 39], this gap is critical as different sectors have their own set of unique challenges and opportunities. We also review the mediating impact of green intrinsic motivation and individual environmental behavioral norms on the CSR-ESBE linkage. Our findings thus delineate the role played by such personality-based variables as mediators between organizational CSR policies and green employee behaviors. This perspective of our study introduces a new insight into the psychological basis of sustainability at workplaces which, until now, have hardly been taken into account within developing countries.

Furthermore, our research links CSR initiatives with the UN-SDGs framework, specifically paying attention to environmental sustainability related goals. The perspective gives insight on a new way of looking at how CSR strategies can dovetail into universal sustainability goals which are an imperative consideration in times of globalization. Finally, our research in this context provides insights into challenges faced by developing economy seeking sustainable development in hospitality and tourism. The geographical focus also contributes significantly to the literature on sustainability research by offering localized and actionable insights across different countries of the world.

## 2. Literature review and hypotheses development

The value-belief-norm (VBN) theory serves as a critical theoretical framework for understanding and explaining human environmental behavior [40]. Developed by Stern and colleagues [41], the VBN theory postulates that individual values influence beliefs, which in turn shape personal norms and consequently drive environmental behavior. This sequence: from values → beliefs → norms→ and finally to behavior forms the core pathway that delineates how individuals process information and make decisions concerning the environment. The VBN theory is manifest in its capacity to illuminate the hidden psychological processes that spur individuals to partake in environmentally friendly actions [42]. It accounts for the multifaceted relationship between personal values, such as those that are altruistic and biospheric, guiding a person’s views concerning the environment. These environmentally oriented beliefs lead to an awareness of the potential consequences and an acceptance of personal responsibility, which then give rise to personal norms, like the inner sense of moral duty to take action. Utilizing VBN theory in the scope of this investigation provides a discerning outlook on how CSR and ESBE, influenced by green intrinsic motivation and personal environmental norms are connected.

Within corporate structures, CSR endeavors mirror wider social and environmental principles, falling in line with the UN-SDGs. VBN theory serves as a crucial tool in decoding how these value-infused initiatives can be converted into individual convictions and stances toward sustainability [43]. These beliefs foster green intrinsic motivation among employees, representing an internal desire to act sustainably. Personal environmental norms, highlighted in VBN theory, act as psychological guides, aligning personal values with organizational CSR efforts and directing employees’ energy-specific behaviors. The VBN theory explains how values, beliefs, and norms culminate in specific behaviors, in this case, ESBE. Employees, motivated by their values and beliefs, and guided by personal environmental norms, engage in energy-specific practices that are in congruence with the organization’s CSR strategies and sustainability goals. In summary, the VBN theory offers a robust theoretical framework that encapsulates the sophisticated network of values, beliefs, norms, and behaviors that shape employees’ reactions to CSR. By exploring this connection through the lens of VBN, this study aims to probe into the psychological roots of ESBE, unearthing the underlying mechanisms that link organizational commitments to individual action. It enhances our understanding of how to foster a work culture that not only supports sustainability at an organizational level but also is in congruence with the personal convictions and values of employees, all in alignment with UN-SDGs.

### 2.1 CSR and energy-specific sustainable behavior of employees

A critical review of existing literature identifies a foundational understanding of the synergy between CSR and ESBE in the context of sustainable development and environmental stewardship [44–46]. While the essence of CSR, as an embodiment of an organization’s commitment to ecological well-being, is somewhat clear [10, 47], a crucial gap emerges when it comes to discerning how this commitment translates into tangible, energy-specific behaviors among employees. Organizations undertake various CSR actions, from emission reduction to community engagement [48–50]. However, the literature often stops short of examining the nuances of how these actions impact the cognitive and behavioral facets of the employees. What remains underexplored is the means by which the broader CSR ethos is distilled down into day-to-day energy conservation habits and the promotion of renewable energy at the workplace [51].

Recent studies have made strides in demonstrating the correlation between CSR and responsible behaviors of employees [52, 53]. Yet, they often paint a broad stroke, overlooking the intricacies of how CSR activities actually seed and nurture ESBE. The repeatedly observed positive correlation between CSR and ESBE in prior studies [54] raises more questions than it answers: How exactly does the broader organizational commitment to CSR engender ESBE? Does the nature and depth of CSR activities have varying impacts on ESBE? These are the gaps this study seeks to fill. In the context of the overarching theory, if CSR is the strategic framework organizations deploy, then ESBE is the operational output manifested through employees. Understanding this relationship at a granular level is indispensable for organizations aiming to maximize their sustainability efforts. In light of the discussed gaps and the theoretical context, our research aims to contribute a deeper understanding to the CSR-ESBE nexus. This culminates in our first hypothesis:

**H1:** CSR is positively related to energy-specific sustainable behavior of employees.

### 2.2 CSR and green intrinsic motivation

The confluence of CSR and green intrinsic motivation offers a sophisticated perspective for organizational strategies interfacing with individual values and beliefs [55]. At the heart of this nexus lies a fundamental question: How do organizational initiatives dovetail with the personal drivers of an employee, especially in the realm of environmental consciousness? Green intrinsic motivation emerges as a potent inner drive crafted from personal norms, values, and beliefs, compelling individuals toward eco-friendly actions [56]. The organizational emphasis on CSR, especially in the environmental dimension, sends a clear message to the workforce: a delineation of values that resonate deeply with the green intrinsic drive [57]. Thus, when employees perceive an alignment between the corporate stance on sustainability and their personal beliefs, it amplifies their green intrinsic motivation [58], a phenomenon that has far-reaching practical implications.

For organizations, particularly in sectors like tourism and hospitality where environmental impact is profound, this alignment is not just beneficial but essential. An engaged and motivated workforce, driven by green intrinsic values, becomes a powerful catalyst in the successful implementation of CSR strategies [59]. The tangible outcome? Enhanced environmental stewardship and a workforce deeply committed to sustainable practices. Peeling back another layer, the VBN theory offers a structured lens to comprehend this CSR and green intrinsic motivation relationship. The VBN theory emphasizes ecological worldviews and the awareness of consequences, and elements mirrored in robust CSR initiatives. These initiatives, when underpinned by the VBN theory, underscore a collective ecological consciousness, making employees more attuned to the implications of their actions [60]. The culmination of these personal values into environmental norms becomes the bridge linking organizational and individual aspirations for sustainability [61]. It is within this theoretical framework that our research positions the intricate dance between CSR and green intrinsic motivation, using the VBN theory as its scaffold. The intertwining of these elements—the organization’s commitment to CSR, an individual’s green intrinsic motivation, and the theoretical insights from VBN—crafts a comprehensive understanding of sustainable behavior.

**H2:** CSR is positively related to the green intrinsic motivation of employees.

### 2.3 Green intrinsic motivation and energy-specific sustainable behavior of employees

The relationship between green intrinsic motivation and ESBE is deeply intertwined with the theoretical foundations of the VBN theory [62]. Green intrinsic motivation is deeply rooted in the value aspect of the VBN theory, signifying the internal ethics and principles that propel individuals toward eco-friendly practices [63]. These profound values induce individuals to act in a manner that mirrors their care for the environment, irrespective of external rewards or pressures [64]. When applied within an organizational setting, green intrinsic motivation manifests in actions that support energy sustainability, forming an integral part of ESBE. Similarly, in the framework of the VBN theory, green intrinsic motivation acts as a bridge between a person’s values and the precise norms steering their actions [65]. These norms, as delineated by the VBN theory, are born from the comprehension of one’s actions’ environmental repercussions, a personal sense of accountability, and the realization of one’s capacity to induce change. When synchronized with green intrinsic motivation, these norms galvanize employees to energetically participate in energy-conservation measures and champion renewable energy within the organization [66].

The link between green intrinsic motivation and ESBE, viewed through the VBN theory’s perspective, aligns with broader international sustainability aspirations, such as the UN-SDGs. By following their intrinsic drive to economize energy and endorse responsible practices, individuals are making a direct contribution to overarching targets like affordable and clean energy (Goal 7) and climate action (Goal 13) Tian. An organizational focus on energy conservation and renewable energy resources can be perceived as a continuation of the VBN framework, where the corporate values resonate with the employees, nurturing a culture of sustainability [67]. This harmony cultivates a communal dedication to energy sustainability, amplifying the resonance of individual contributions and thereby fostering an environment that enables collective progress towards a more sustainable future.

With this theoretical underpinning, the third hypothesis of this study is framed:

**H3:** Green intrinsic motivation of employees is positively related to ESBE.

### 2.4 Mediating function of green intrinsic motivation

The interplay between CSR and ESBE is intricate, revealing a multifaceted dynamic mediated by green intrinsic motivation. At the forefront, CSR initiatives serve as a beacon of an organization’s ethos and dedication toward environmental sustainability [68]. Employees, upon perceiving these initiatives, might experience an amplification or ignition of their green intrinsic motivation [69]. Essentially, the alignment between individual convictions and organizational commitment crystallizes into a potent force for environmental stewardship [70]. This motivational catalyst operates as a bridge, channeling the organization’s high-level CSR values into concrete actions exemplified in ESBE. This bridging phenomenon can be understood deeply when underpinned by the VBN theory [71]. The VBN theory delineates a clear trajectory from personal values to pro-environmental behavior via environmental beliefs and norms. Therefore, CSR, as an embodiment of organizational commitment to the environment, synergizes with individual employee values. This harmonious convergence invigorates green intrinsic motivation, nurturing an organizational milieu that champions and incentivizes sustainable energy behaviors [72].

But this is not just about organizations and their immediate workforce. This mediation mechanism resonates with larger global paradigms, specifically the UN-SDGs—particularly Goals 7 (Affordable and Clean Energy) and 13 (Climate Action). By fostering green intrinsic motivation, companies not only align their CSR strategies with ESBE but also plug into the broader global ambition of sustainable energy and climate action. Beyond strategy, understanding the psychological levers, like intrinsic motivation, that influence individual behavior becomes paramount. In doing so, organizations are better poised to transform their CSR endeavors into tangible energy-saving actions, culminating in a genuine organizational culture of sustainability. Synthesizing these insights, our study postulates:

**H4:** Green intrinsic motivation mediates the positive relationship between CSR and ESBE.

### 2.5 CSR and personal environmental norms and energy-specific sustainable behavior of employees

CSR initiatives symbolize an organization’s commitment to sustainability and environmental protection [73, 74], mirroring the core principles found in many of the UN SDGs [75]. When these CSR activities align with employees’ personal environmental norms, the connection is more profound, leading to more significant engagement in sustainable practices [76]. Personal environmental norms, shaped by individual values and beliefs, often direct behavior, guiding choices towards eco-friendly actions [77]. CSR efforts can, therefore, act as a catalyst, reinforcing or even shaping these norms. The VBN theory provides a solid theoretical underpinning for this relationship. It emphasizes the role of values, beliefs, and norms in guiding human behavior. Within the organizational context, the expression of values through CSR activities can be seen as an external manifestation of what many individuals might hold as personal values [43]. When these align, the CSR activities not only reflect the corporate stance on environmental issues but also resonate with employees’ personal environmental norms, reinforcing their existing beliefs and encouraging the adherence to these norms.

From the perspective of the UN-SDGs, particularly Goals 12 (responsible consumption and production), 13 (climate action), and 15 (life on land), the alignment of CSR with personal environmental norms contributes to a global endeavor to promote sustainable living and environmental stewardship [78, 79]. Employees’ adherence to personal environmental norms, encouraged by CSR, plays a vital role in reducing negative environmental impacts, conserving natural resources, and promoting responsible consumption.

This interconnection between CSR and personal environmental norms signifies a multilayered relationship where organizational values are not only reflected in company policies but also embodied by individual employees in their daily decisions and actions. Recognizing and leveraging this link can enable organizations to foster a culture of environmental responsibility that aligns with both individual norms and global sustainability objectives [77]. From these insights, the fifth hypothesis of this study is formulated as follows:

**H5:** CSR positively influences the personal environmental norms of employees.

### 2.6 Personal environmental norms and energy-specific sustainable behavior of employees

Personal environmental norms act as an individual’s internal ethical compass, guiding their daily decisions and actions toward environmental sustainability. These norms can be analyzed through the VBN theory, which maps the journey from personal values to beliefs and, ultimately, to norms that direct human conduct. In a workplace scenario, these norms have a significant influence on the environmentally responsible behavior of individuals [80]. The practice of ESBE within an organization can be perceived as a tangible reflection of personal environmental norms, as employees convert their individual dedication to environmental care into actionable measures in their professional environment [81].

The criticality of this connection is further emphasized by alignment with the UN-SDGs. Specific objectives such as Goal 7, Goal 12 and Goal 13 inherently resonate with the concept of ESBE [82]. The adherence of employees to these personal environmental norms, followed by their active efforts towards energy preservation and sustainable practices, directly facilitates the accomplishment of these global targets. By adhering to personal environmental norms that are in congruence with ESBE, employees can actively contribute to organizational sustainability. This connection reinforces the idea that individual actions are not isolated but part of a broader effort to promote responsible energy consumption and production. It amplifies the role of personal responsibility in shaping a sustainable future, emphasizing that individual decisions and behaviors are pivotal in achieving larger organizational and global sustainability goals. The sixth hypothesis of this study, deeply rooted in the perspectives of UN-SDGs and VBN, is thus articulated as follows:

**H6:** Personal environmental norms of employees positively influence ESBE.

### 2.7 The mediating function of personal environmental norms

CSR, with its ethos rooted in ethical and sustainable practices, indisputably impacts the propensity for ESBE within organizations [15]. However, the empirical literature suggests a nuanced pathway of this influence, where personal environmental norms act as a critical intermediary [83]. Understanding this mediation is not just essential for establishing a direct linkage between CSR and ESBE but also offers a granular insight into the cognitive processes of employees, bridging their individual ethos with organizational initiatives [77]. The VBN theory provides a comprehensive scaffold to elucidate this mediation [84]. While CSR symbolizes the organizational values, it’s the employees’ interpretation of these values, and the subsequent formation of beliefs about sustainability that cultivates personal environmental norms. These norms, in essence, crystallize the employee’s cognitive journey from acknowledging organizational values to translating them into tangible behaviors like ESBE [85]. Thus, the profound intersection of CSR and personal environmental norms underscores the potential for organizations to magnify ESBE by targeting and nurturing these norms.

Moreover, by recognizing and tapping into this linkage, organizations are not just tailoring initiatives in isolation. They’re also contributing to wider global sustainability targets. There’s a profound realization that the amplification of ESBE is not solely a product of organizational values but rather a harmonization of these values with individual norms. In essence, an alignment strategy creates a feedback loop: CSR initiatives resonate with employees, reinforcing personal norms, which in turn drive ESBE. Existing literature offers insights into the effects of CSR and the importance of ESBE but falls short of exploring the intricate interplay with personal environmental norms. This study seeks to address this lacuna, presenting an enhanced theoretical framework that integrates CSR, personal environmental norms, and ESBE. Consequently, our study posits:

**H7:** Personal environmental norms of employees mediate the relationship between CSR and ESBE.

**Fig 1:**
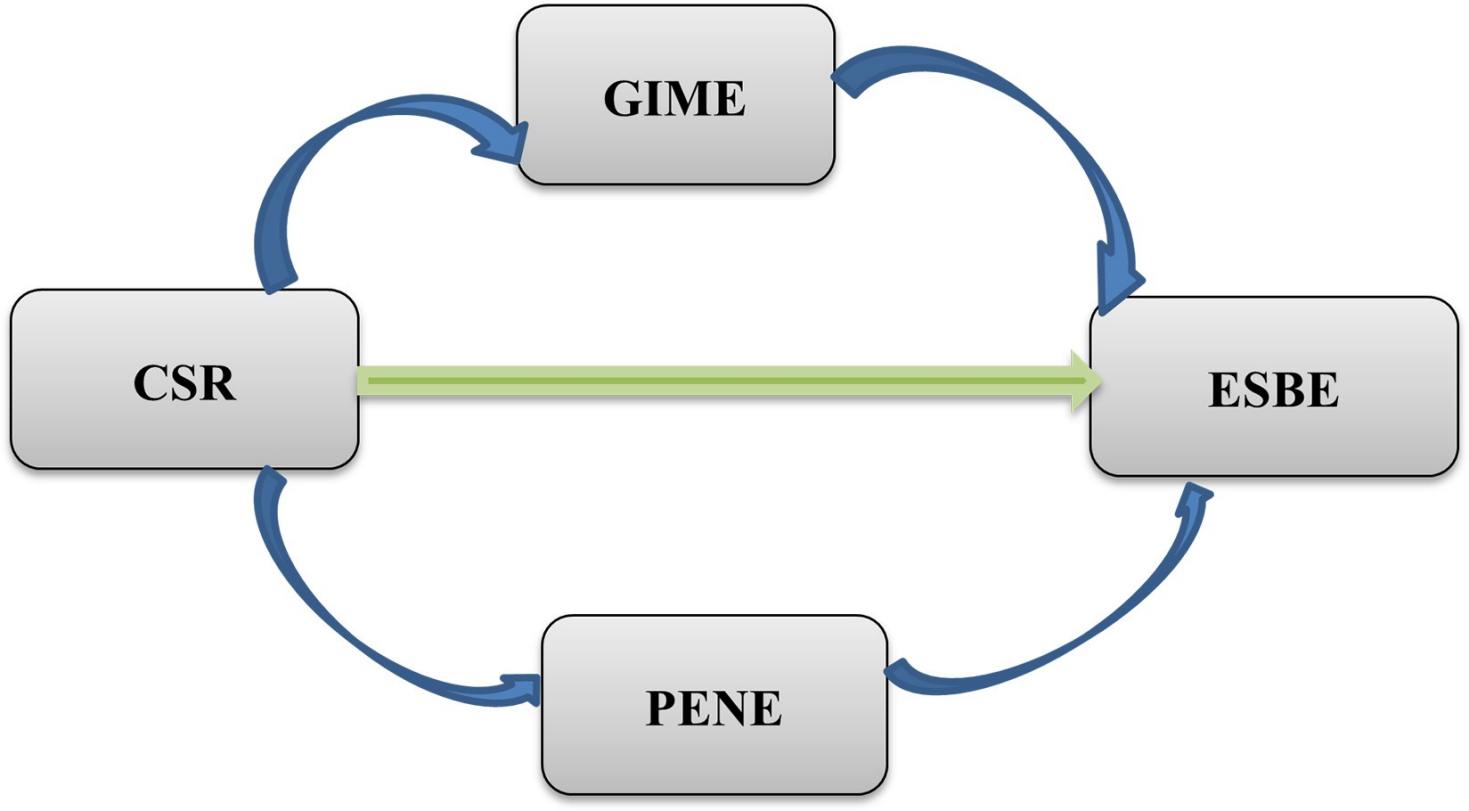
The research model of this study: CSR = corporate social responsibility, GIME = green intrinsic motivation of employees, PENE = personal environmental norms of employees, ESBE = energy-specific sustainable behavior of employees.

Fig 1 represents the research model of the current study.

## 3. Methodology

The tourism and hospitality sector of Pakistan has witnessed remarkable growth in recent years, reflecting a surge in both domestic and international tourism [33]. With its rich cultural heritage, diverse landscapes, and expanding urban centers, Pakistan has become an attractive destination. Specifically, the upscale hotel chains in the country have played a significant role in this growth, showing visible CSR engagement to uplift communities and protect the natural environment [6]. The growth in the sector has environmental implications. Notably, it has a pronounced carbon footprint, which has a marked effect on environmental health and climate dynamics. For our research, we zeroed in on Pakistan’s hospitality arena, specifically highlighting upscale hotels because of their evident CSR actions. We focused on Lahore, Karachi, and Islamabad, given their susceptibility to changing weather patterns. Both Lahore and Karachi grapple with significant environmental challenges, such as heightened pollution levels, urban temperature spikes, and coastal wear [31, 86]. Our decision to spotlight upscale hotel chains in Pakistan had both data-driven and pragmatic underpinnings. On one hand, these chains serve as a snapshot of CSR actions, giving deep insights into sustainability efforts. On the other, their substantial carbon emissions make them an ideal lens to view the broader role of CSR in the hospitality world. These issues are intensified by swift urban growth and shifting climate patterns, positioning these cities as essential focal points to explore the relationship between CSR, green-driven motivation, individual environmental standards, and ESBE.

In our selection of Lahore, Karachi, and Islamabad as the primary cities for our study, we aimed to capture a cross-section of Pakistan’s rapidly expanding hospitality sector while also acknowledging the environmental challenges these urban areas face. The upscale hotels in these cities not only provide tangible CSR initiatives but also reside in cities grappling with significant environmental concerns. By focusing on these urban hubs, we’re effectively studying the CSR initiatives in areas where their impact, both positive and negative, is likely the most pronounced. These locations allow us to capture the dynamics of CSR and environmental challenges, providing a rich context for understanding how CSR might drive green intrinsic motivation and ESBE among employees.

To ensure a comprehensive understanding of the subject, we requested different upscale hospitality organizations to facilitate us in the data collection activity, aligning with the larger interests of industry and academia. Out of numerous requests, six hotels responded positively, allowing us to gather data from various employees serving in these organizations. Employees in the hospitality sector were purposefully selected to take part in this study. Their experiences and perspectives, being directly involved in implementing sustainability practices, contributed instrumental information regarding the execution of CSR initiatives within the industry. Our participant selection from employees was similarly reasoned. Employees, especially in customer-facing roles, are at the forefront of implementing and translating CSR initiatives into actual practice. By capturing their perceptions and experiences, we could gain insights into the gaps between organizational intent and on-the-ground reality.

Ethics were at the forefront of the research process, with careful adherence to the Helsinki Declaration’s guidelines to uphold ethical standards [87]. The study commenced by obtaining written informed consent from each participating employee [2]. This document transparently delineated the study’s purpose, the nature of participation, measures to maintain confidentiality, and the unconditional right to withdraw from the study without any consequences. This strategy not only confirmed that participation was voluntary but also cultivated an atmosphere of trust and openness. A strict commitment to anonymity was maintained, ensuring that all personal and identifiable information remained separate from the collected responses. Data was managed with the highest level of confidentiality, restricted solely to the research team’s access, and securely stored to ward off unauthorized access. Additionally, the research design and its execution were scrutinized by an autonomous ethical review board to guarantee alignment with both local and global ethical standards. These procedures went beyond merely fulfilling ethical guidelines; they embodied a profound dedication to honoring the participants’ autonomy, dignity, and privacy. By cultivating an ethical research milieu, the study assured that the insights obtained were not only instrumental in understanding CSR in the hospitality realm but were also garnered in a way that steadfastly respected the highest ethical principles.

The data collection was structured into three waves, separated by an interval of two weeks. In the first wave, we concentrated on the personal environmental norms of employees and their perception of CSR in their organizations. The second wave focused on the employees’ green intrinsic motivation. In the final wave, we collected data related to ESBE. We distributed a total of 500 questionnaires in our survey. Following the first wave of distribution, we received 420 fully completed questionnaires, translating to an initial response rate of 84%. In the second wave, the number of fully filled questionnaires dropped to 380, bringing the cumulative response rate down to 76%. By the end of the third and final wave, we garnered 354 fully completed questionnaires, which pegged the final cumulative response rate at 70.8%. Upon excluding partially completed or invalid questionnaires, we were left with 325 valid responses, giving us an overall valid response rate of 65%.

The three-wave data collection approach was strategically designed to reduce potential bias and response fatigue. Spreading the data collection process over three distinct waves allowed us to dive deep into each set of variables, personal environmental norms, perception of CSR, green intrinsic motivation, and ESBE, ensuring that participants had ample time to reflect on each topic and provide thoughtful responses. This phased approach also minimized the chances of respondents being influenced by their answers to previous questions, leading to more reliable data.

### 3.1 Estimation of sample size

The process of selecting an appropriate sample is pivotal to ensuring the robustness, statistical power, and validity of a study’s outcomes, especially when utilizing methods like structural equation modeling (SEM) that examine intricate relationships among numerous variables. In this research, we opted for the a-priori method for determining our sample size. This method is not only well-regarded for its rigorous approach and scientific accuracy but has also been acknowledged and endorsed by several researchers for its appropriateness in sample size estimation [86, 88, 89]. By using the a-priori method, we were equipped to pre-determine the minimal sample size required to detect a particular effect size with a defined level of confidence prior to initiating data collection. This preemptive strategy ensures that our study holds adequate power to reveal noteworthy relationships among the investigated variables.

The research included four principal variables: CSR with six items, green intrinsic motivation of employees (GIME) with six items, personal environmental norms of employees (PENE) with four items, and ESBE with eight items. Given the multifaceted nature and the number of items correlated with these variables, the a-priori sample size calculator explicitly designed for SEM was used.

The significance of using this calculator for Partial Least Squares Structural Equation Modeling (PLS-SEM) is paramount. PLS-SEM is a sophisticated analytical technique [90], and determining a suitable sample size manually can be exceptionally challenging. This calculator takes into account various statistical characteristics exclusive to SEM, enabling an accurate and customized estimation that accommodates the particular structure and prerequisites of the model. Based on the entered parameters, including the number of variables, corresponding items, and specific statistical considerations, the a-priori calculator suggested a recommended sample size of 286 for the research.

The demographic composition of our sample reflects the diversity and inclusivity of the research design. Participants comprised both male and female employees from different departments within the selected hospitality organizations, representing a varied range of ages and experiences. Out of the total valid responses (n = 325), a substantial proportion of the participants were male (73%), in line with the gender distribution in the workplace in Pakistan’s hospitality sector, a male-dominant society. The remaining 27% of participants were female. Participants were drawn from several key departments within the hotel chains, encompassing Housekeeping, Food and Beverage Service, Front Office, Human Resources, and General administration, to name a few. This diversity in departmental representation ensured that the study captured a broad spectrum of experiences and perspectives of employees. Moreover, employees of varied age groups and with diverse experience were invited to partake in the study. In addition to the diverse departmental representation, participants were also categorized based on their job levels within the hotel chains. We ensured to include various job levels ranging from management to supervisory roles, as well as frontline and support staff. This added dimension allowed for an understanding of the potential differences or similarities in perceptions and behaviors across different hierarchical levels. Specifically, participants included room staff, support staff, restaurant staff, and other service providers. Understanding the job roles of participants not only enriches the context of the study but provides deeper insights into the attitudes and behaviors of different employee categories in relation to CSR initiatives.

Regarding our sample’s demographic composition, the observed gender distribution is reflective of the broader gender dynamics within the hospitality sector in Pakistan. In a male-dominated society [91], the workplace often mirrors societal norms, which, in this case, is a male-majority representation. By capturing this distribution, our research accurately reflects the real-world scenario, adding another layer of authenticity and relevance to our findings. Table 1 provides more detail on the demographic details of our sample.

**Table 1:** Demographic composition of respondents (N = 325).

Category	Sub-Category	Number of Respondents	Percentage (%)	Cumulative Number of Respondents	Cumulative Percentage (%)
**Gender**	Male	237	73	237	73
	Female	88	27	325	100
**Department**	Housekeeping	55	17	55	17
	Food and Beverage Service	70	21.5	125	38.5
	Front Office	65	20	190	58.5
	Human Resources	40	12.3	230	70.8
	General Administration	95	29.2	325	100
**Job Level**	Management	75	23	75	23
	Supervisory	85	26.2	160	49.2
	Room Staff	55	17	215	66.2
	Support Staff	60	18.5	275	84.7
	Restaurant Staff	50	15.4	325	100

### 3.2 Method bias and social desirability

Common method bias (CMB) and social desirability are critical issues that can compromise the integrity of research findings, particularly in behavioral studies [92, 93]. To mitigate these concerns in the present study, several theoretical and empirical steps were implemented. Anonymity was assured to encourage truthful responses and minimize the tendency to answer in a socially desirable manner. The likelihood of respondents falling into a pattern of answering, a common source of CMB, was reduced by varying the order of questions. A time lag between measurements was introduced, collecting data in three waves separated by an interval of two weeks, to minimize the effects of transient mood states or environmental conditions that could contribute to CMB. Careful wording and pretesting of the questionnaire ensured that the items were clear and unambiguous, reducing potential social desirability bias. The study utilized established and validated measures that have shown resistance to both CMB and social desirability. Empirically, Harman’s single-factor test was used to detect any manifestation of CMB. This test involves conducting a factor analysis to see if a single factor emerges or if one general factor accounts for the majority of the covariance between the measures. The results of the test in this study indicated the non-existence of any CMB issue, adding confidence to the integrity of the study’s findings. The combination of these theoretical precautions and empirical validation ensured that the relationships between the variables were not unduly influenced by the method of measurement itself, strengthening the validity and reliability of the study’s results.

### 3.3 Instrument

In the research conducted, information was collected through a modified questionnaire, employing the traditional paper-and-pencil technique. This approach was intentionally selected to foster a setting that promoted genuine answers and helped increase participation among workers in the hospitality sector. The decision to employ adapted scales was a carefully considered one, based on the knowledge of their proven validity and dependability in previous instances. The use of such thoroughly validated scales ensured that the concepts under examination were measured with both accuracy and precision, thus bolstering the overall trustworthiness of the information gathered. Furthermore, the choice to modify existing scales reflected a commitment to upholding scientific standards, leveraging insights from earlier empirical research and foundational theories. Moreover, the choice to use the traditional paper-and-pencil method for data collection added a tangible and personable dimension to the process, a factor that is often appreciated by respondents.

Specifically, the predictor variable was (CSR), measured by adapting six items from Turker [94] scale. This scale is renowned for assessing CSR perceptions among employees, with sample items such as ‘This hospitality organization implements special programs to minimize its negative impact on the natural environment.’ ESBE was the criterion variable for the survey. We adapted eight electricity-related items from the study by Blok, Wesselink [9] to suit the context of this research, specifically focusing on behaviors related to heating or cooling, lighting, and computer use. A representative item from this scale is ‘When I leave my office for a considerable period of time, and there is no one else.’

Two intervening variables were included in this survey: PENE and GNIM. PENE was measured using four items from the study by Nasir Ansari and Irfan [77], which included illustrative statements like ‘I feel personally obliged to save as much environmental degradation as possible.’ Meanwhile, GNIM was assessed using six items adapted from Li, Bhutto [22], originally based on the study by Blok, Wesselink [9]. A sample item from this scale is ‘I enjoy trying to solve environmental tasks on the job.’

## 4. Results

### 4.1 Initial analysis

**Fig 2:**
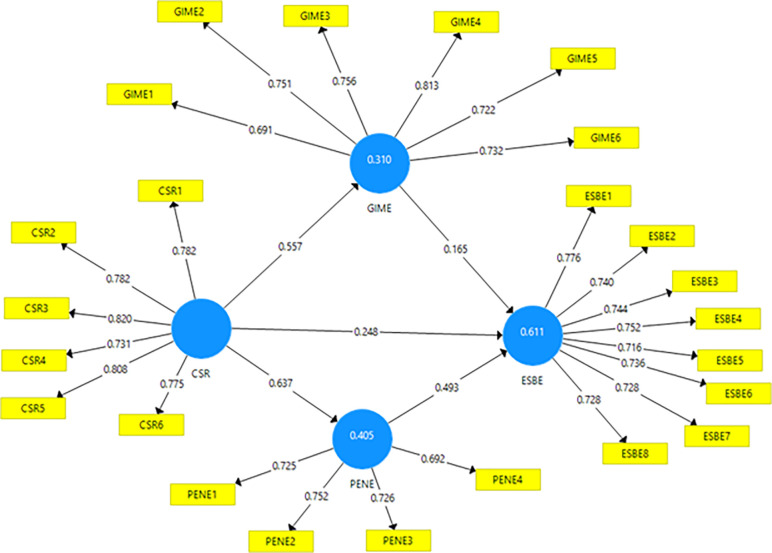
The measurement model of the current study.

Using SMART-PLS software, confirmatory factor analysis (CFA) was executed to determine the reliability and validity of constructs. Factor loadings for CSR, ESBE, GIME, and PENE are respectively reported, with the T Statistics for all variables falling between 12.6 to 36.2. See Table 2 and Fig 2 for detailed results. Construct reliability and validity were gauged through measures like Cronbach’s alpha, rho_A, CR, and AVE. All constructs showed significant internal consistency and convergent validity, with values consistently surpassing accepted benchmarks. Results are consolidated in Table 3.

**Table 2:** Output of CFA.

	Original Sample (O)	Sample Mean (M)	Standard Deviation (STDEV)	T Statistics (|O/STDEV|)
**CSR1 <—CSR**	0.782	0.783	0.027	28.982
**CSR2 <—CSR**	0.782	0.78	0.026	29.848
**CSR3 <—CSR**	0.820	0.82	0.023	36.2
**CSR4 <—CSR**	0.731	0.729	0.036	20.26
**CSR5 <—CSR**	0.808	0.807	0.023	35.143
**CSR6 <—CSR**	0.775	0.774	0.024	32.443
**ESBE1 <—ESBE**	0.776	0.775	0.026	29.834
**ESBE2 <—ESBE**	0.740	0.742	0.059	12.6
**ESBE3 <—ESBE**	0.744	0.744	0.025	30.121
**ESBE4 <—ESBE**	0.752	0.754	0.034	21.974
**ESBE5 <—ESBE**	0.716	0.717	0.036	19.89
**ESBE6 <—ESBE**	0.736	0.735	0.028	26.478
**ESBE7 <—ESBE**	0.728	0.726	0.035	20.606
**ESBE8 <—ESBE**	0.728	0.728	0.028	25.844
**GIME1 <—GIME**	0.691	0.693	0.039	17.771
**GIME2 <—GIME**	0.751	0.749	0.033	23.034
**GIME3 <—GIME**	0.756	0.755	0.029	25.695
**GIME4 <—GIME**	0.813	0.813	0.024	34.527
**GIME5 <—GIME**	0.722	0.72	0.038	19.132
**GIME6 <—GIME**	0.732	0.731	0.039	18.813
**PENE1 <—PENE**	0.725	0.725	0.034	21.166
**PENE2 <—PENE**	0.752	0.754	0.029	25.804
**PENE3 <—PENE**	0.726	0.727	0.039	18.411
**PENE4 <—PENE**	0.692	0.692	0.036	19.056

**Table 3:** Reliability and validity.

	Cronbach’s Alpha	rho_A	CR	AVE
**CSR**	0.874	0.876	0.905	0.614
**ESBE**	0.882	0.883	0.906	0.548
**GIME**	0.839	0.843	0.882	0.555
**PENE**	0.700	0.704	0.815	0.524

The correlation matrix (Table 4) showed discriminant validity for each construct. For instance, CSR had a diagonal value of 0.783, surpassing off-diagonal correlations, illustrating its discriminant validity. This pattern was consistent across other constructs. The HTMT ratio of correlations demonstrated discriminant validity. All HTMT ratios were below the benchmark of 0.85 [95], ensuring distinct phenomena for the constructs, as seen in Table 5.

**Table 4:** Correlations and discriminant validity.

	CSR	ESBE	GIME	PENE
**CSR**	0.783			
**ESBE**	0.653	0.74		
**GIME**	0.557	0.542	0.745	
**PENE**	0.637	0.731	0.485	0.724

**Table 5:** Heterotrait-Monotrait (HTMT) ratio of correlations.

	CSR	ESBE	GIME	PENE
**CSR**				
**ESBE**	0.74			
**GIME**	0.646	0.626		
**PENE**	0.803	0.811	0.627	

### 4.5 Structural analysis results

In this analysis, SEM was employed to test the hypotheses which is an advanced level data analysis tool [96–98]. Direct relationships were observed across several variables. The relationship between CSR and ESBE shows a coefficient of 0.248 with a statistically significant t-value of 3.905 and a p-value of 0.000, indicating a direct positive relationship. Similarly, the relationship between CSR and GIME is substantial, with a coefficient of 0.557 and a significant t-value of 8.763. The relationship between CSR and PENE is also strong, evidenced by a coefficient of 0.637 and a t-value of 16.795. GIME’s direct effect on ESBE and PENE’s direct effect on ESBE are also marked, with t-values of 3.042 and 9.043, respectively. These findings confirm the statistical significance of H1, H2, H3, H5, and H6.

**Fig 3:**
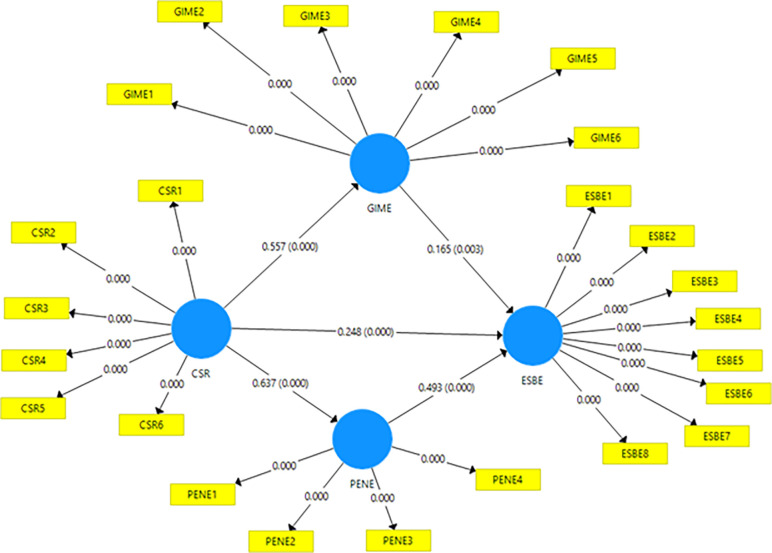
The Structural model of the current study.

Indirect effects were also found to be significant. The indirect influence of CSR on ESBE through GIME is demonstrated by a coefficient of 0.092 and a t-value of 2.738, with a p-value of 0.006. Similarly, the relationship between CSR and ESBE via PENE has a coefficient of 0.314 and a t-value of 7.041, highlighting the significant indirect pathways. Hence, H4, and H7 were statistically significant. Table 6 and Fig 3 summarize the structural model analysis.

**Table 6:** Structural analysis summary.

	(O)	(M)	(SD)	*t*	*p*	2.50%	97.50%
**CSR -> ESBE**	0.248	0.247	0.064	3.905	0.000	0.119	0.366
**CSR -> GIME**	0.557	0.556	0.064	8.763	0.000	0.421	0.671
**CSR -> PENE**	0.637	0.642	0.038	16.795	0.000	0.545	0.701
**GIME -> ESBE**	0.165	0.162	0.054	3.042	0.002	0.062	0.285
**PENE -> ESBE**	0.493	0.498	0.054	9.043	0.000	0.38	0.598
**CSR -> GIME -> ESBE**	0.092	0.091	0.034	2.738	0.006	0.034	0.172
**CSR -> PENE -> ESBE**	0.314	0.32	0.045	7.041	0.000	0.239	0.398

## 5. Discussion and conclusion

The results obtained through our research, contribute with important information on the CSR and ESBE within the tourism and hospitality sector in Pakistan. Particularly, our study validates the important effect that CSR has on ESBE. Like other scholars [52, 53] have pointed out, this research emphasizes ethics in business practice and organizational integrity as crucial drivers of ESBE in an emerging market environment. Our research shows that organizations should align their CSR strategies with employee’s green intrinsic motivations and link it to their personal values. This is a significant finding especially to developing countries such as Pakistan, where most of the time environmental problems are closely related to social and economic dilemmas. As such, CSR programs should align with employees’ intrinsic motivation to foster sustainability initiatives.

Secondly, our study supports the fact that intrinsic values can also be strong motivators for eco-friendly practices at work [58]. Our research in this regard enhances the comprehension of how personal values and organizational policies can interact together in order to make environment friendly practices successful. We have discovered a novel perspective on CSR and green intrinsic motivation as enacted toward the employees of an organization. Such a perception can assist organizations to come up effective CSR schemes that go along with the interests of personnel without necessarily compromising environmental protection. By encouraging their employees to get involved in sustainable practices, it could bring about an enthusiastic workforce that will drive sustainability internally. Our study contributes a new outlook towards the linkage between personal environmental norms, CSR, and the resulting ESBE. Our research contributes within the ongoing discourse on how organizations can incorporate their personal value systems with cultural value systems, strategic planning and operation.

Our research concludes that CSR, green intrinsic motivation, personal environmental norms, and ESBE are inextricably linked. We offer empirical evidence towards Pakistan’s tourism and hospitality industry towards promoting CSR as a measure of achieving environmental sustainability in developing countries. The findings demonstrate the importance of matching organization strategies with staff motivations as well as social norms for sustainable success.

### 5.1 Implications

Besides being a theoretical contribution, our study makes a meaningful contribution both locally and globally, especially in Pakistan’s tourism and hospitality sector. Firstly, our results highlight the importance of CSR in enhancing ESBE. The fact that such a move is necessary has been shown in our study. Companies, particularly in the sector of tourism and hospitality, have to consider CSR as part of their corporate goals because it builds a culture of sustainable practices. This is among the ways through which they integrate CSR to their day-to-day operations like coming up with specific CSR policies that directly focus on environmental preservation and energy conservation.

The second consideration in the study relates to the match between company CSR initiatives and employee environmental (green) motivations. The employees may be motivated to act in an enthusiastic manner as well as protect the environment. Therefore, organizations should think of instituting training programs and workshops which will enlighten the employees on the need for sustainable activities and what they should do to support this exercise. Our study further highlights the significance of CSR initiatives that align with UN-SDGs. The SDGs can be used as guidelines to design organization’s CSR activities to make them support global sustainability and increase the organizations’ international goodwill. It also emphasizes the situation in Pakistan, an underdeveloped country that has its own peculiarities in culture, economics, and society. With regards to Pakistan, the initiatives of CSR should be adapted to conform to local sensitivities so as to make them culturally appropriate. For example, CSR actions within the tourism field can be centered on preserving cultural heritages, community based tourism and educating locals about environment conservation.

Furthermore, our research implies that the CRS should be personalized towards employee’s environment. By capitalizing on the different individual motivations, values and perspectives of workforce, organizations can adopt a much broader approach towards sustainability. Lastly, our study focuses on energy specific behavior which can be used as a template organization’s targeted strategy towards energy management. This is even more important in countries such as Pakistan where there are significant problems with regards to the supply of energy. Utilization of energy efficient practices and technologies by institutions from tourism and hospitality sectors help to minimize their carbon footprints as well as cut down on production cost.

Based on this study, we offer the following recommendations:

- Formulate CSR policies aimed at environmental protection and conservation of energy.
- Provide employees with training in sustainability.
- Structure the CSR activities around the UN SDGs.
- Embed regular educational and training initiatives centered on sustainable methodologies into operational frameworks.
- Champion collaborations bridging governmental sectors, non-governmental organizations, and industry leaders, fostering a united front for sustainable progression and preservation

### 5. 2. Limitations and future research directions

The potential limitations of our study are its narrow data collection from only three cities and six hotels, thus potentially affecting the validity of the results. Tourists as well as local communities’ perspectives were not considered in this regard. While the cross-sectional design has a snapshot of the time, a longitudinal approach can be employed to reveal underlying trends. In spite of strong ethical and methodological considerations, with a 65% response rates there may exist non-response bias, while self-reported data can be subjective.

Therefore, further studies are required to extend these relationships beyond narrower spaces involving different stakeholders, with particular emphasis on specific CSR programs within the unique Pakistani environmental and cultural backdrop. The research could unveil more universal sustainability practices, if it compared such practices at upscale hotels with small or rural accommodation establishments. Longitudinal studies might help us understand how CSR initiatives develop, as well as the evolution of environmental concerns. Future research should include application of the Delphi Method that integrates both quantitative and qualitative methods, particularly on policy making. Conducting additional research and looking into other important tourist and hotel outlets could shed more light on this aspect.

## Supporting information

S1 Appendix (DOCX)
