# Supplementary material for: Green sustainability in the hotel sector: The role of CSR, intrinsic green motivation, and personal environmental norms
Source: PLoS One. 2024 Jun 27;19(6):e0295850. doi: 10.1371/journal.pone.0295850 (PMC11210877; doi:10.1371/journal.pone.0295850)
Supplement: S1 Appendix — (DOCX) [file pone.0295850.s001.docx]

**Appendix 1:** The Questionnaire used in this survey

| **Corporate Social Responsibility** | | | | |  | | | |  |
| --- | --- | --- | --- | --- | --- | --- | --- | --- | --- |
| **Question** | | **Strongly Disagree (1)** | **Disagree (2)** | **Neutral (3)** | **Agree (4)** | **Strongly Agree (5)** | | | |
| This hospitality organization participates in activities that aim to protect and improve the quality of the natural environment | |  |  |  |  | | | |  |
| This hospitality organization makes investments to create a better life for future generations | |  |  |  |  | | | |  |
| This hospitality organization implements special programs to minimize its negative impact on the natural environment | |  |  |  |  | | | |  |
| This hospitality organization targets sustainable growth, which considers future generations | |  |  |  |  | | | |  |
| This hospitality organization supports non-governmental organizations that work in problematic areas | |  |  |  |  | | | |  |
| This hospitality organization contributes to the campaigns and projects that promote the well-being of society | |  |  |  |  | | | |  |
| **Energy-specific Sustainable Behavior of Employees** | | | | | | | | | |
| **Question** | **Strongly Disagree (1)** | | **Disagree (2)** | **Neutral (3)** | **Agree (4)** | | | **Strongly Agree (5)** | |
| I check whether thermostats are set correctly in my office. |  | |  |  |  | | | |  |
| I wear more/less clothes instead of putting the heating/cooling on. |  | |  |  |  | | | |  |
| I make sure that heating/air conditioning is off or reduced outside working hours. |  | |  |  |  | | | |  |
| I switch off or reduce heating/air conditioning in unused rooms |  | |  |  |  | | | |  |
| I switch on the lights when I come to the office in the morning and switch them off when I leave |  | |  |  |  | | | |  |
| When I leave my office for a considerable period, and there is no one else, I switch off the lights |  | |  |  |  | | | |  |
| I switch off my computer/notebook when I leave my office for a considerable period |  | |  |  |  | | | |  |
| I switch off my computer/notebook when I go home |  | |  |  |  | | | |  |
| **Green Intrinsic Motivation** | | | | |  | | | |  |
| **Question** | **Strongly Disagree (1)** | | **Disagree (2)** | **Neutral (3)** | **Agree (4)** | | **Strongly Agree (5)** | | |
| I feel personally obliged to save as much environmental degradation as possible |  | |  |  |  | | | |  |
| I feel morally obliged to save the environment, regardless of what others do |  | |  |  |  | | | |  |
| I feel morally obliged to use green instead of regular electricity |  | |  |  |  | | | |  |
| I feel obliged to bear the environment and nature in mind in my daily behavior |  | |  |  |  | | | |  |
|  |  | |  |  |  | | | |  |
| **Personal Environmental Norms** | | | | |  | | | |  |
| **Question** | **Strongly Disagree (1)** | | **Disagree (2)** | **Neutral (3)** | **Agree (4)** | | **Strongly Agree (5)** | | |
| I enjoy coming up with new green ideas |  | |  |  |  | | | |  |
| I enjoy trying to solve environmental tasks on the job |  | |  |  |  | | | |  |
| I enjoy tackling environmental tasks that are completely new |  | |  |  |  | | | |  |
| I enjoy improving existing green ideas at the job |  | |  |  |  | | | |  |
| I feel excited when I have new green ideas |  | |  |  |  | | | |  |
| I feel like becoming further engaged in the development of green ideas |  | |  |  |  | | | |  |
